# Supplementary material for: Longitudinal Correlations between Molecular Compositions of Stratum Corneum and Breast Milk Factors during Infancy: A Prospective Birth Cohort Study
Source: Nutrients. 2024 Jun 16;16(12):1897. doi: 10.3390/nu16121897 (PMC11206726; doi:10.3390/nu16121897)
Supplement: Supplementary file 1 [file nutrients-16-01897-s001.zip › nutrients-3015965-supplementary.pdf]

Supplementary materials

Figure S1

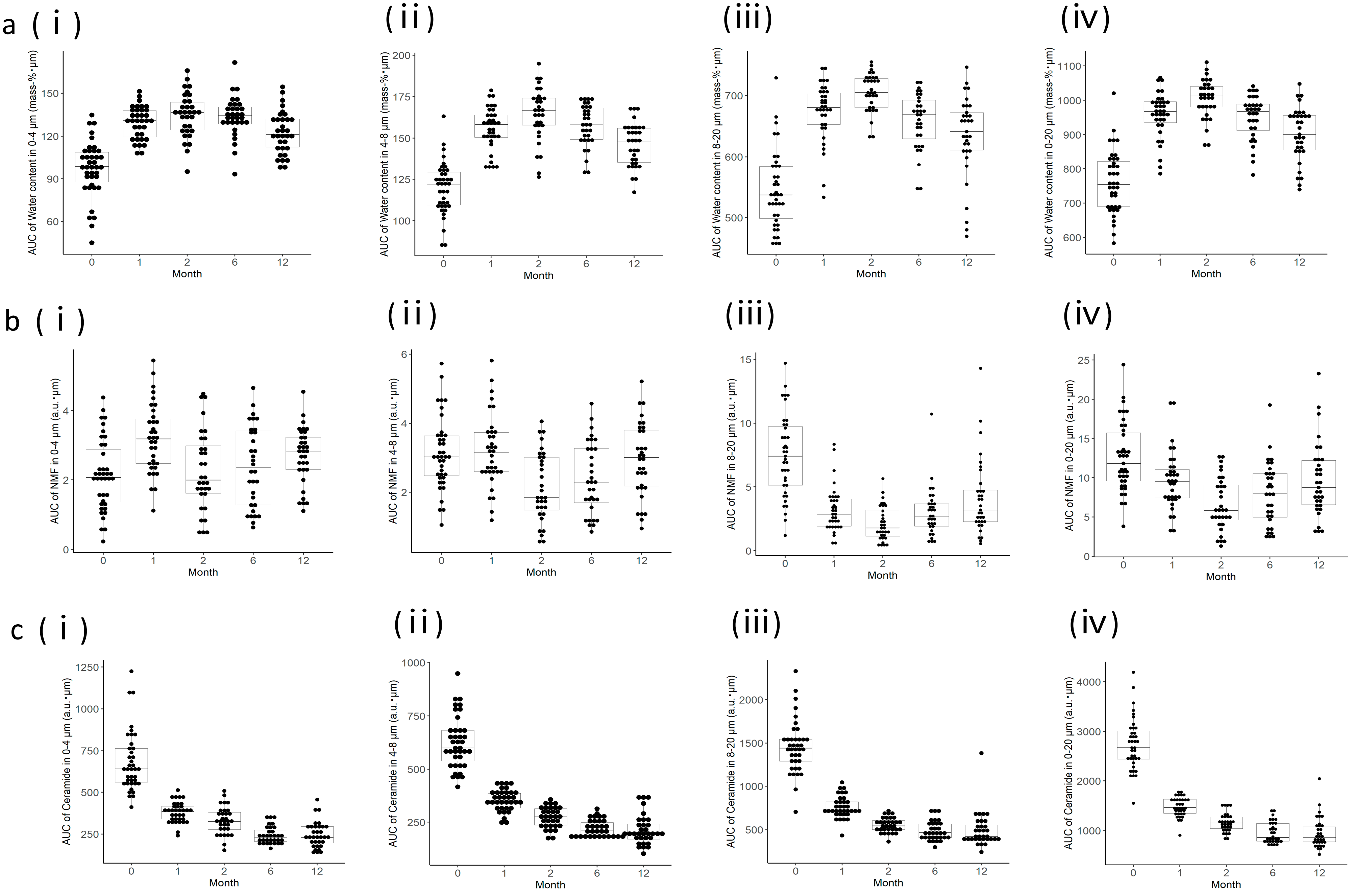

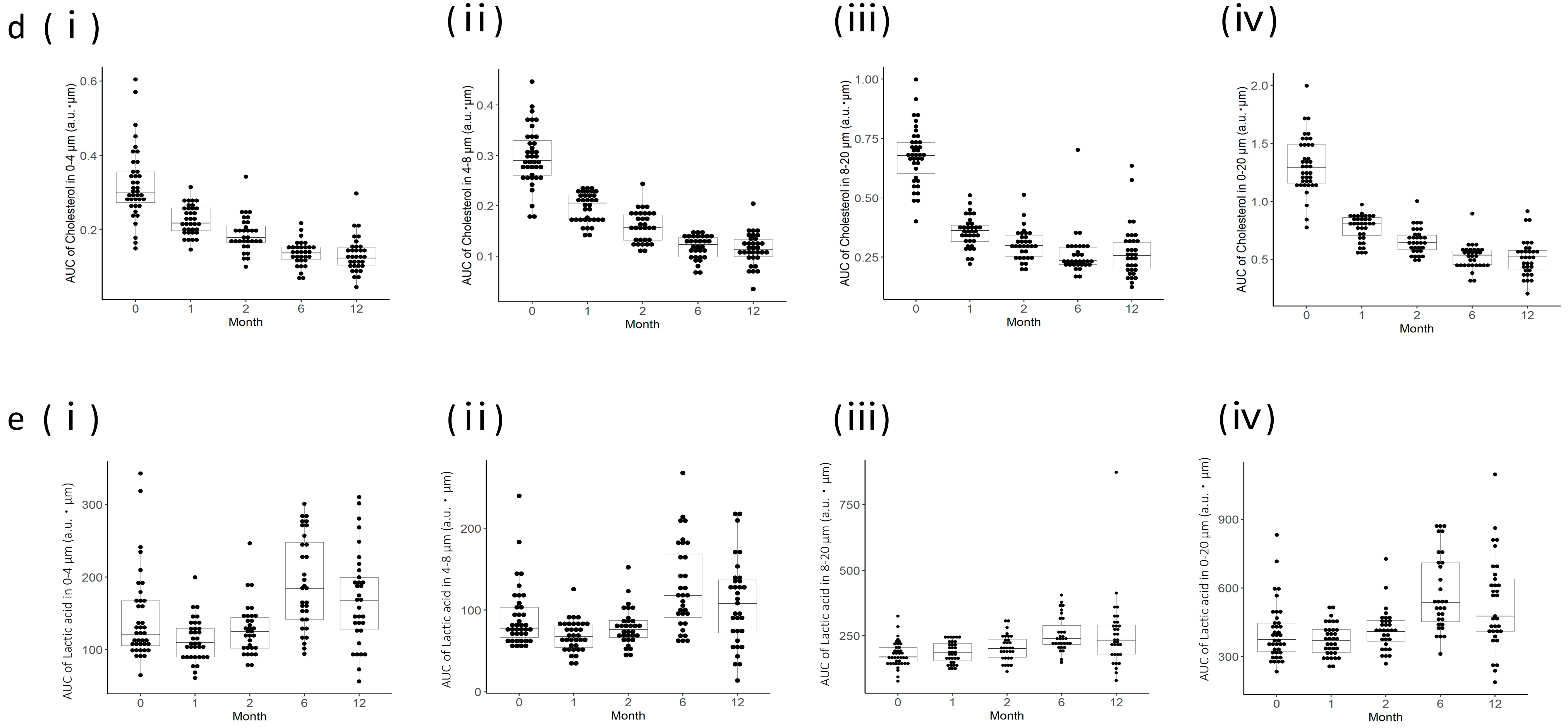

Figure S1 Changes in the median area under the curve (AUC) for molecular composition of the stratum corneum (SC) measured by a confocal Raman spectrometer (CRS) over time

Median AUC values for water content (a), natural moisturizing factor (NMF) (b), ceramide (c), cholesterol (d), and lactic acid (e) at each month of age as measured by a confocal Raman spectrometer (CRS) are shown for every 0-4 (i), 4-8 (ii), 8-20 (iii), and 0-20 (iv) am. Box plots represent medians with 25th and 75th percentile values, min–max range, and outliers. In the AUCs for lactic acid (8-20 μm and 0-20 μm), outliers (8-20 μm: 3373.456, 0-20 μm: 3843.873) at 6 months for the same patient were excluded. As shown in Figure 1, the mean of five measurements was considered as each participant's representative value.

Figure S2

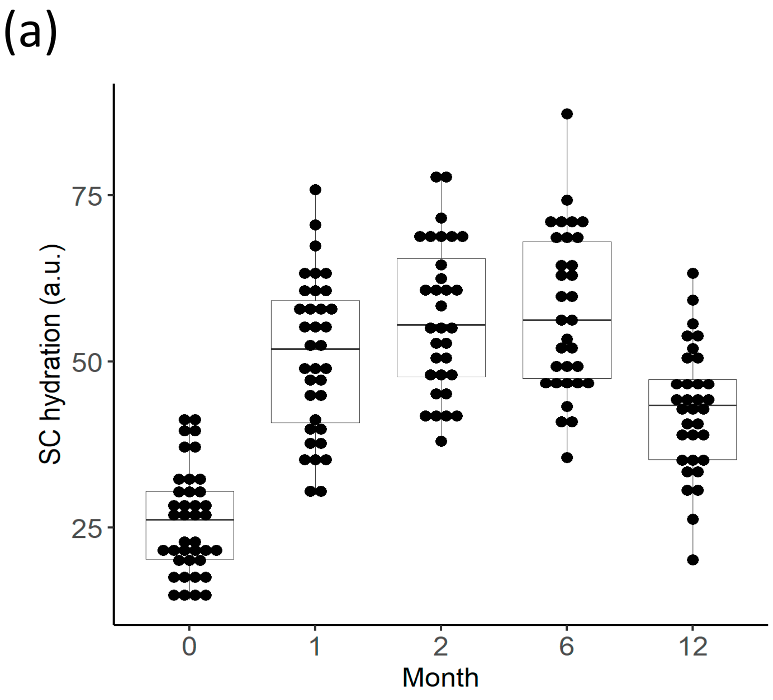

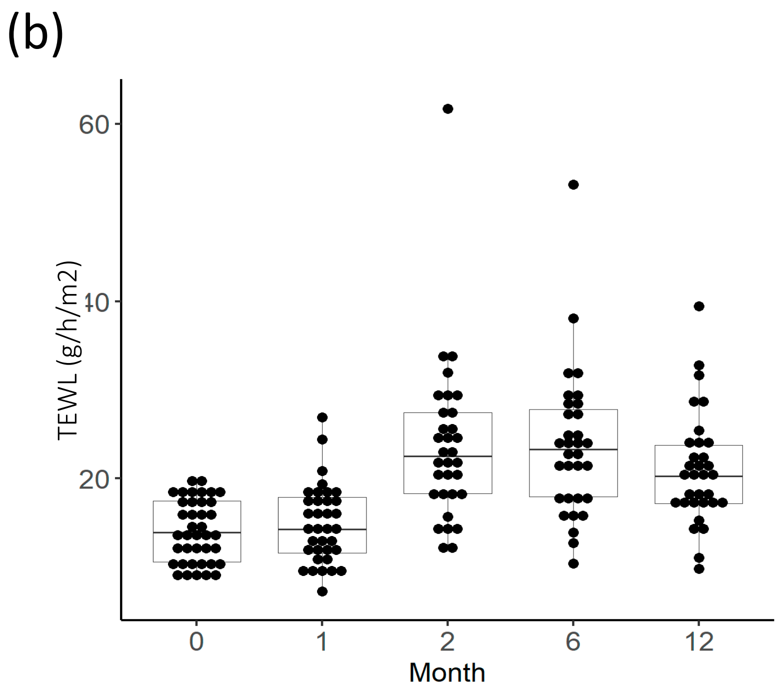

Figure S2 Changes in median values of stratum corneum (SC) hydration and transepidermal water content (TEWL) over time

Median values of SC hydration (a) and TEWL (b) are shown at 0, 1, 2, 6, and 12 months of age. Tewameter measurements were performed once on the calf, and measurements using a corneometer were performed five times, with a representative value shown. For an explanation of the box plots, see the Legend of Figure 2.

Figure S3

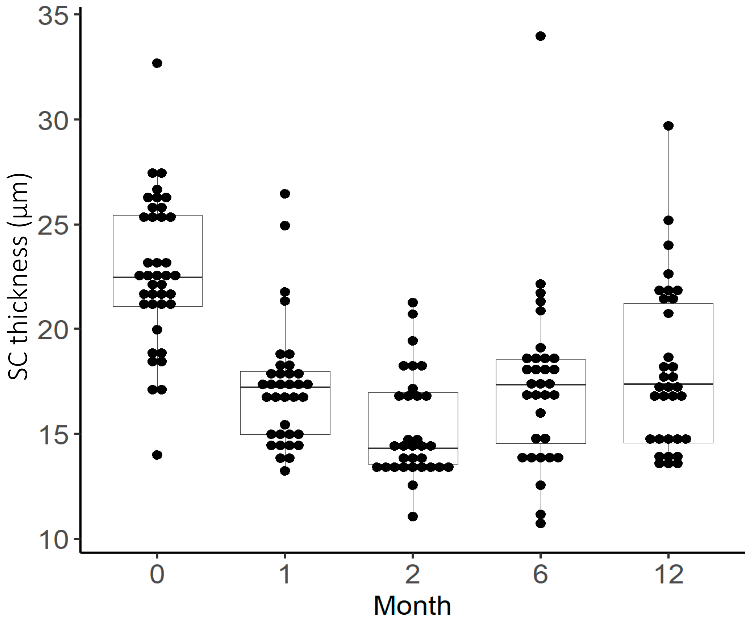

Figure S3 Changes in stratum corneum (SC) thickness

Median values for SC thickness at each month of age are shown. For an explanation of the box plots, see the Legend of Figure 2.

Figure S4

a

Water content		Breast milk factors															
depth	month	TGF-β1 (pg./ml)				TGF-β2 (pg./ml)				IgA (mg/ml)				Lactoferrin (mg/ml)			
		0	1	2	6	0	1	2	6	0	1	2	6	0	1	2	6
0-4	0	-0.326 (-0.582 - -0.012)*				-0.353 (-0.602 - -0.043)*				-0.335 (-0.588 - -0.021)*				-0.201 (-0.485 - 0.123)			
	1	-0.364 (-0.622 - -0.035)*	-0.263 (-0.548 - 0.077)			-0.394 (-0.643 - -0.070)*	-0.161 (-0.469 - 0.182)			-0.184 (-0.487 - 0.159)	-0.048 (-0.375 - 0.290)			-0.046 (-0.373 - 0.292)	-0.006 (-0.339 - 0.328)		
	2	-0.144 (-0.469 - 0.215)	-0.135 (-0.462 - 0.224)	-0.002 (-0.351 - 0.347)		-0.183 (-0.500 - 0.177)	-0.059 (-0.399 - 0.296)	0.239 (-0.120 - 0.542)		-0.084 (-0.421 - 0.272)	-0.015 (-0.362 - 0.335)	-0.081 (-0.418 - 0.276)		0.183 (-0.177 - 0.500)	0.100 (-0.258 - 0.434)	-0.086 (-0.422 - 0.271)	
	6	-0.035 (-0.379 - 0.317)	-0.037 (-0.398 - 0.334)	0.092 (-0.299 - 0.456)	-0.059 (-0.399 - 0.296)	-0.006 (-0.354 - 0.343)	-0.136 (-0.479 - 0.242)	0.158 (-0.237 - 0.507)	-0.198 (-0.511 - 0.162)	-0.085 (-0.421 - 0.272)	0.014 (-0.355 - 0.378)	-0.195 (-0.536 - 0.199)	0.125 (-0.234 - 0.454)	-0.010 (-0.357 - 0.340)	0.080 (-0.295 - 0.434)	-0.337 (-0.636 - 0.049)	-0.227 (-0.533 - 0.132)
	12	-0.081 (-0.412 - 0.270)	-0.010 (-0.369 - 0.352)	0.089 (-0.302 - 0.453)	-0.148 (-0.494 - 0.238)	0.042 (-0.306 - 0.380)	-0.155 (-0.488 - 0.218)	-0.016 (-0.394 - 0.366)	-0.298 (-0.604 - 0.084)	0.079 (-0.272 - 0.411)	0.243 (-0.129 - 0.554)	0.049 (-0.337 - 0.422)	-0.053 (-0.417 - 0.327)	0.285 (-0.064 - 0.573)	0.247 (-0.125 - 0.557)	0.179 (-0.216 - 0.523)	0.044 (-0.335 - 0.410)
4-8	0	-0.348 (-0.598 - -0.036)*				-0.368 (-0.612 - -0.059)*				-0.405 (-0.639 - -0.102)*				-0.230 (-0.509 - 0.092)			
	1	-0.357 (-0.617 - -0.027)*	-0.174 (-0.479 - 0.169)			-0.412 (-0.655 - -0.091)*	-0.025 (-0.355 - 0.311)			-0.240 (-0.531 - 0.101)	-0.211 (-0.509 - 0.131)			-0.150 (-0.460 - 0.193)	-0.150 (-0.460 - 0.193)		
	2	-0.100 (-0.434 - 0.257)	-0.077 (-0.414 - 0.280)	0.106 (-0.252 - 0.438)		0.029 (-0.323 - 0.374)	-0.052 (-0.393 - 0.302)	0.176 (-0.184 - 0.495)		-0.143 (-0.468 - 0.217)	-0.077 (-0.415 - 0.279)	-0.086 (-0.422 - 0.271)		0.163 (-0.196 - 0.485)	0.211 (-0.149 - 0.521)	-0.012 (-0.360 - 0.338)	
	6	-0.123 (-0.452 - 0.236)	-0.115 (-0.462 - 0.262)	-0.099 (-0.462 - 0.292)	-0.150 (-0.474 - 0.210)	-0.210 (-0.521 - 0.149)	-0.110 (-0.458 - 0.267)	-0.087 (-0.452 - 0.303)	-0.326 (-0.605 - 0.026)	-0.271 (-0.566 - 0.086)	-0.074 (-0.429 - 0.301)	-0.170 (-0.517 - 0.224)	-0.085 (-0.421 - 0.272)	-0.174 (-0.493 - 0.186)	-0.140 (-0.482 - 0.238)	-0.185 (-0.528 - 0.210)	-0.224 (-0.531 - 0.135)
	12	-0.194 (-0.503 - 0.160)	-0.102 (-0.446 - 0.268)	-0.063 (-0.433 - 0.324)	-0.122 (-0.473 - 0.264)	-0.043 (-0.381 - 0.305)	-0.103 (-0.447 - 0.267)	-0.125 (-0.482 - 0.268)	-0.338 (-0.632 - 0.040)	-0.263 (-0.556 - 0.089)	0.130 (-0.242 - 0.468)	-0.128 (-0.485 - 0.265)	-0.216 (-0.545 - 0.171)	0.009 (-0.335 - 0.352)	0.077 (-0.292 - 0.425)	0.015 (-0.367 - 0.393)	-0.136 (-0.485 - 0.249)
8-20	0	-0.074 (-0.380 - 0.248)				-0.070 (-0.377 - 0.251)				-0.348 (-0.598 - -0.036)*				-0.360 (-0.606 - -0.050)*			
	1	-0.206 (-0.505 - 0.136)	-0.090 (-0.411 - 0.250)			-0.188 (-0.490 - 0.155)	-0.010 (-0.342 - 0.325)			-0.115 (-0.432 - 0.227)	-0.130 (-0.444 - 0.212)			-0.175 (-0.480 - 0.168)	-0.042 (-0.370 - 0.295)		
	2	-0.027 (-0.373 - 0.324)	-0.025 (-0.370 - 0.327)	0.226 (-0.133 - 0.533)		0.129 (-0.230 - 0.457)	-0.136 (-0.462 - 0.224)	0.162 (-0.197 - 0.484)		-0.069 (-0.408 - 0.287)	-0.220 (-0.528 - 0.139)	-0.159 (-0.481 - 0.201)		0.212 (-0.148 - 0.522)	0.129 (-0.230 - 0.457)	-0.070 (-0.408 - 0.286)	
	6	-0.265 (-0.562 - 0.093)	-0.254 (-0.568 - 0.124)	-0.284 (-0.599 - 0.108)	-0.239 (-0.543 - 0.119)	-0.371 (-0.637 - -0.026)*	-0.199 (-0.527 - 0.181)	-0.206 (-0.543 - 0.189)	-0.413 (-0.666 - -0.075)*	-0.024 (-0.370 - 0.327)	-0.049 (-0.408 - 0.324)	-0.072 (-0.440 - 0.317)	-0.085 (-0.422 - 0.271)	-0.286 (-0.577 - 0.069)	-0.258 (-0.571 - 0.120)	-0.182 (-0.526 - 0.213)	-0.294 (-0.583 - 0.061)
	12	-0.143 (-0.464 - 0.210)	-0.251 (-0.560 - 0.120)	-0.159 (-0.508 - 0.235)	-0.194 (-0.529 - 0.193)	0.099 (-0.253 - 0.427)	-0.124 (-0.463 - 0.247)	-0.029 (-0.404 - 0.355)	-0.331 (-0.627 - 0.048)	-0.256 (-0.551 - 0.096)	-0.054 (-0.406 - 0.312)	-0.268 (-0.588 - 0.125)	-0.170 (-0.511 - 0.217)	-0.110 (-0.437 - 0.242)	-0.034 (-0.389 - 0.331)	-0.293 (-0.606 - 0.098)	-0.384 (-0.662 - -0.013)*
0-20	0	-0.172 (-0.462 - 0.152)				-0.187 (-0.474 - 0.137)				-0.404 (-0.638 - -0.102)*				-0.349 (-0.599 - -0.038)*			
	1	-0.199 (-0.499 - 0.144)	-0.084 (-0.406 - 0.256)			-0.227 (-0.521 - 0.115)	-0.018 (-0.349 - 0.317)			-0.134 (-0.448 - 0.208)	-0.140 (-0.452 - 0.203)			-0.141 (-0.453 - 0.202)	-0.030 (-0.360 - 0.306)		
	2	-0.061 (-0.401 - 0.294)	-0.088 (-0.424 - 0.269)	0.172 (-0.188 - 0.491)		0.113 (-0.245 - 0.444)	-0.087 (-0.423 - 0.270)	0.181 (-0.179 - 0.498)		-0.153 (-0.476 - 0.207)	-0.168 (-0.488 - 0.192)	-0.137 (-0.463 - 0.223)		0.163 (-0.197 - 0.484)	0.200 (-0.160 - 0.513)	-0.061 (-0.401 - 0.294)	
	6	-0.201 (-0.513 - 0.159)	-0.227 (-0.548 - 0.152)	-0.195 (-0.535 - 0.200)	-0.241 (-0.544 - 0.117)	-0.254 (-0.554 - 0.104)	-0.169 (-0.504 - 0.211)	-0.178 (-0.523 - 0.216)	-0.397 (-0.655 - -0.056)*	-0.121 (-0.451 - 0.238)	-0.157 (-0.495 - 0.223)	-0.197 (-0.537 - 0.198)	-0.110 (-0.441 - 0.249)	-0.197 (-0.511 - 0.163)	-0.254 (-0.567 - 0.124)	-0.269 (-0.589 - 0.123)	-0.337 (-0.614 - 0.013)
	12	-0.150 (-0.469 - 0.204)	-0.206 (-0.527 - 0.167)	-0.138 (-0.492 - 0.255)	-0.189 (-0.525 - 0.198)	0.108 (-0.244 - 0.435)	-0.094 (-0.439 - 0.276)	-0.041 (-0.414 - 0.344)	-0.380 (-0.659 - -0.008)*	-0.311 (-0.591 - 0.036)	0.061 (-0.306 - 0.412)	-0.160 (-0.509 - 0.234)	-0.181 (-0.519 - 0.206)	-0.079 (-0.411 - 0.271)	0.048 (-0.318 - 0.401)	-0.211 (-0.547 - 0.184)	-0.299 (-0.605 - 0.083)

Diff: 1.0, 0.8, 0.6, 0.4, 0.2, 0, -0.2, -0.4, -0.6, -0.8, -1.0

Figure S4 Spearman's Correlation coefficient of molecular components in the stratum corneum (SC) measured by confocal Raman spectroscopy and breast milk factors for each month of age

Correlation coefficients and 95% confidence intervals between the median area under the receiver operating curve by depth (0–4, 4–8, 8–20, and 0–20 μm) for water content (**a**), and natural moisturizing factor (NMF) (**b**), ceramide (**c**), cholesterol (**d**), lactic acid (**e**) and breast milk factors (TGF-β1.2, IgA, lactoferrin) at each month of age are shown; $p < 0.05^{*}$. Moisture content, ceramide, and cholesterol tended to be negatively correlated with all breast milk factors during the entire period, while NMF and lactoferrin were positively correlated with all breast milk factors.

b

NMF		Breast milk factors															
depth	month	TGF-β1 (pg./ml)				TGF-β2 (pg./ml)				IgA (mg/ml)				Lactoferrin (mg/ml)			
		0	1	2	6	0	1	2	6	0	1	2	6	0	1	2	6
0-4	0	-0.116 (-0.416 - 0.207)				-0.089 (-0.394 - 0.233)				-0.346 (-0.596 - -0.034)*				0.107 (-0.216 - 0.409)			
	1	-0.084 (-0.406 - 0.257)	-0.099 (-0.419 - 0.242)			-0.116 (-0.432 - 0.226)	0.001 (-0.332 - 0.334)			0.034 (-0.302 - 0.364)	0.092 (-0.249 - 0.412)			0.301 (-0.036 - 0.576)*	-0.098 (-0.418 - 0.243)		
	2	0.050 (-0.304 - 0.392)	0.078 (-0.279 - 0.415)	-0.270 (-0.565 - 0.087)		-0.047 (-0.389 - 0.307)	0.213 (-0.147 - 0.523)	-0.156 (-0.478 - 0.204)		-0.064 (-0.404 - 0.291)	0.104 (-0.254 - 0.437)	0.030 (-0.322 - 0.375)		-0.020 (-0.366 - 0.331)	-0.083 (-0.419 - 0.274)	0.026 (-0.326 - 0.371)	
	6	-0.105 (-0.438 - 0.253)	-0.133 (-0.476 - 0.245)	-0.182 (-0.526 - 0.213)	0.008 (-0.342 - 0.355)	-0.054 (-0.395 - 0.300)	0.249 (-0.129 - 0.564)	0.207 (-0.188 - 0.544)	0.239 (-0.120 - 0.542)	-0.127 (-0.455 - 0.232)	0.099 (-0.278 - 0.449)	0.190 (-0.205 - 0.532)	0.133 (-0.226 - 0.461)	0.264 (-0.093 - 0.561)	0.202 (-0.177 - 0.530)	0.361 (-0.022 - 0.651)	0.350 (0.002 - 0.623)
	12	0.099 (-0.253 - 0.428)	0.256 (-0.115 - 0.564)	-0.064 (-0.434 - 0.324)	0.039 (-0.339 - 0.407)	-0.097 (-0.426 - 0.255)	0.462 (0.122 - 0.705)*	0.108 (-0.284 - 0.469)	0.436 (0.075 - 0.696)*	0.053 (-0.296 - 0.389)	0.122 (-0.250 - 0.462)	0.232 (-0.162 - 0.562)	-0.014 (-0.385 - 0.361)	0.038 (-0.310 - 0.376)	0.011 (-0.350 - 0.370)	0.340 (-0.046 - 0.638)	0.189 (-0.198 - 0.525)
4-8	0	-0.061 (-0.369 - 0.260)				-0.077 (-0.383 - 0.245)				-0.332 (-0.586 - -0.018)*				0.143 (-0.181 - 0.438)			
	1	-0.078 (-0.401 - 0.262)	-0.091 (-0.412 - 0.250)			-0.112 (-0.429 - 0.230)	0.006 (-0.328 - 0.338)			0.125 (-0.217 - 0.440)	0.156 (-0.187 - 0.465)			0.356 (0.026 - 0.616)*	-0.129 (-0.443 - 0.214)		
	2	0.052 (-0.302 - 0.394)	0.014 (-0.336 - 0.361)	-0.309 (-0.594 - 0.044)		-0.070 (-0.409 - 0.285)	0.171 (-0.189 - 0.491)	-0.086 (-0.422 - 0.271)		-0.069 (-0.408 - 0.287)	0.169 (-0.191 - 0.489)	0.065 (-0.290 - 0.405)		-0.014 (-0.361 - 0.336)	-0.100 (-0.434 - 0.258)	0.121 (-0.238 - 0.451)	
	6	0.069 (-0.287 - 0.408)	-0.043 (-0.403 - 0.329)	-0.073 (-0.441 - 0.316)	0.116 (-0.242 - 0.447)	0.096 (-0.261 - 0.431)	0.219 (-0.161 - 0.542)	0.258 (-0.135 - 0.581)	0.322 (-0.030 - 0.603)	-0.183 (-0.500 - 0.177)	0.096 (-0.280 - 0.447)	0.179 (-0.216 - 0.523)	0.152 (-0.207 - 0.476)	0.167 (-0.193 - 0.487)	0.184 (-0.196 - 0.516)	0.284 (-0.108 - 0.599)	0.312 (-0.041 - 0.596)*
	12	0.020 (-0.325 - 0.361)	0.212 (-0.161 - 0.532)	-0.097 (-0.460 - 0.294)	0.093 (-0.291 - 0.450)	-0.175 (-0.489 - 0.179)	0.286 (-0.083 - 0.586)	0.068 (-0.320 - 0.437)	0.378 (0.005 - 0.658)*	0.139 (-0.215 - 0.460)	0.248 (-0.124 - 0.558)	0.345 (-0.040 - 0.641)	0.223 (-0.164 - 0.550)	0.143 (-0.211 - 0.463)	-0.021 (-0.379 - 0.342)	0.446 (0.080 - 0.706)*	0.317 (-0.064 - 0.617)
8-20	0	-0.038 (-0.349 - 0.281)				0.030 (-0.288 - 0.342)				0.088 (-0.234 - 0.392)				0.352 (0.041 - 0.601)*			
	1	-0.111 (-0.428 - 0.231)	-0.045 (-0.373 - 0.293)			-0.109 (-0.426 - 0.233)	0.118 (-0.224 - 0.435)			0.142 (-0.201 - 0.454)	0.208 (-0.134 - 0.506)			0.355 (0.025 - 0.616)*	-0.131 (-0.445 - 0.212)		
	2	-0.061 (-0.401 - 0.294)	0.027 (-0.325 - 0.372)	-0.246 (-0.548 - 0.112)		-0.107 (-0.439 - 0.251)	0.197 (-0.163 - 0.511)	-0.184 (-0.500 - 0.176)		0.009 (-0.341 - 0.357)	0.197 (-0.163 - 0.511)	0.169 (-0.191 - 0.489)		-0.016 (-0.362 - 0.335)	-0.071 (-0.409 - 0.285)	0.196 (-0.164 - 0.510)	
	6	0.102 (-0.256 - 0.435)	0.108 (-0.269 - 0.456)	0.134 (-0.259 - 0.489)	0.351 (0.002 - 0.623)*	0.065 (-0.290 - 0.405)	0.397 (0.035 - 0.666)*	0.298 (-0.093 - 0.609)	0.445 (0.114 - 0.687)*	-0.302 (-0.588 - 0.053)	-0.030 (-0.392 - 0.340)	0.034 (-0.351 - 0.408)	0.152 (-0.207 - 0.476)	0.073 (-0.283 - 0.411)	0.087 (-0.289 - 0.440)	0.164 (-0.230 - 0.512)	0.406 (0.067 - 0.661)*
	12	0.095 (-0.257 - 0.424)	0.284 (-0.085 - 0.585)	0.023 (-0.360 - 0.400)	0.106 (-0.278 - 0.461)	-0.088 (-0.419 - 0.263)	0.356 (-0.005 - 0.635)	0.084 (-0.306 - 0.450)	0.304 (-0.078 - 0.608)	0.060 (-0.289 - 0.396)	0.260 (-0.111 - 0.567)	0.437 (0.068 - 0.701)*	0.109 (-0.275 - 0.463)	0.149 (-0.204 - 0.469)	0.070 (-0.298 - 0.420)	0.517 (0.171 - 0.750)*	0.331 (-0.048 - 0.627)
0-20	0	-0.053 (-0.362 - 0.267)				0.002 (-0.314 - 0.318)				-0.117 (-0.417 - 0.206)				0.303 (-0.014 - 0.564)			
	1	-0.083 (-0.405 - 0.257)	-0.052 (-0.379 - 0.286)			-0.117 (-0.433 - 0.225)	0.089 (-0.252 - 0.410)			0.088 (-0.253 - 0.409)	0.167 (-0.176 - 0.474)			0.365 (0.036 - 0.623)*	-0.110 (-0.427 - 0.232)		
	2	-0.017 (-0.364 - 0.333)	0.063 (-0.292 - 0.403)	-0.257 (-0.556 - 0.101)		-0.057 (-0.398 - 0.298)	0.235 (-0.124 - 0.539)	-0.110 (-0.442 - 0.248)		-0.015 (-0.362 - 0.336)	0.146 (-0.214 - 0.471)	0.087 (-0.270 - 0.423)		0.033 (-0.319 - 0.377)	-0.069 (-0.408 - 0.286)	0.183 (-0.177 - 0.500)	
	6	0.073 (-0.283 - 0.411)	-0.037 (-0.398 - 0.334)	-0.088 (-0.453 - 0.302)	0.113 (-0.245 - 0.444)	0.069 (-0.287 - 0.408)	0.281 (-0.095 - 0.587)	0.180 (-0.215 - 0.524)	0.284 (-0.071 - 0.576)	-0.205 (-0.517 - 0.155)	0.034 (-0.337 - 0.396)	0.103 (-0.288 - 0.465)	0.107 (-0.251 - 0.439)	0.166 (-0.194 - 0.486)	0.167 (-0.213 - 0.502)	0.188 (-0.207 - 0.530)	0.343 (-0.006 - 0.618)
	12	0.110 (-0.242 - 0.437)	0.318 (-0.048 - 0.608)	0.016 (-0.366 - 0.394)	0.121 (-0.264 - 0.473)	-0.095 (-0.424 - 0.257)	0.444 (0.100 - 0.694)*	0.159 (-0.235 - 0.509)	0.439 (0.079 - 0.698)*	0.019 (-0.327 - 0.360)	0.205 (-0.168 - 0.526)	0.413 (0.039 - 0.685)*	0.090 (-0.293 - 0.448)	0.087 (-0.264 - 0.418)	0.059 (-0.308 - 0.410)	0.588 (0.268 - 0.791)*	0.315 (-0.066 - 0.616)

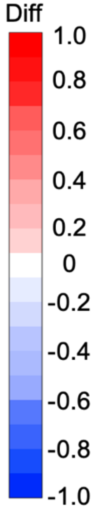

C

Ceramide depth	month	Breast milk factors: TGF-β1 (pg./ml) 0	1	2	6
0-4	0	0.242 (-0.080 - 0.518)			
	1	-0.016 (-0.348 - 0.319)	-0.114 (-0.431 - 0.228)		
	2	0.262 (-0.096 - 0.559)	0.103 (-0.255 - 0.436)	0.041 (-0.312 - 0.384)	
	6	-0.344 (-0.619 - 0.005)	-0.414 (-0.678 - -0.056)*	-0.119 (-0.477 - 0.273)	-0.121 (-0.451 - 0.237)
	12	-0.149 (-0.468 - 0.205)	-0.178 (-0.506 - 0.195)	0.133 (-0.260 - 0.488)	-0.034 (-0.402 - 0.343)
4-8	0	0.166 (-0.158 - 0.458)			
	1	-0.052 (-0.379 - 0.286)	-0.167 (-0.474 - 0.176)		
	2	0.201 (-0.159 - 0.514)	0.151 (-0.209 - 0.475)	-0.092 (-0.427 - 0.266)	
	6	-0.014 (-0.361 - 0.336)	-0.240 (-0.558 - 0.138)	-0.146 (-0.498 - 0.248)	-0.252 (-0.552 - 0.106)
	12	-0.262 (-0.555 - 0.089)	-0.222 (-0.539 - 0.150)	0.050 (-0.336 - 0.422)	0.072 (-0.310 - 0.433)
8-20	0	0.010 (-0.306 - 0.325)			
	1	-0.090 (-0.411 - 0.250)	-0.121 (-0.437 - 0.221)		
	2	0.020 (-0.331 - 0.366)	0.185 (-0.175 - 0.501)	-0.028 (-0.373 - 0.324)	
	6	0.110 (-0.249 - 0.441)	0.015 (-0.354 - 0.379)	0.212 (-0.183 - 0.548)	-0.079 (-0.416 - 0.277)
	12	0.017 (-0.328 - 0.358)	-0.235 (-0.549 - 0.137)	-0.239 (-0.568 - 0.155)	-0.327 (-0.624 - 0.052)
0-20	0	0.083 (-0.239 - 0.388)			
	1	-0.093 (-0.413 - 0.248)	-0.204 (-0.503 - 0.139)		
	2	0.160 (-0.200 - 0.482)	0.245 (-0.114 - 0.547)	0.035 (-0.317 - 0.379)	
	6	-0.051 (-0.393 - 0.303)	-0.169 (-0.505 - 0.210)	0.109 (-0.282 - 0.470)	-0.149 (-0.473 - 0.210)
	12	-0.123 (-0.447 - 0.230)	-0.220 (-0.537 - 0.153)	-0.036 (-0.410 - 0.349)	-0.151 (-0.496 - 0.236)

Ceramide depth	month	TGF-β2 (pg./ml) 0	1	2	6
0-4	0	0.118 (-0.205 - 0.418)			
	1	-0.256 (-0.543 - 0.084)	-0.014 (-0.345 - 0.321)		
	2	0.058 (-0.297 - 0.398)	0.056 (-0.298 - 0.397)	-0.166 (-0.486 - 0.194)	
	6	-0.407 (-0.661 - -0.067)*	-0.305 (-0.604 - 0.069)	0.185 (-0.210 - 0.528)	-0.153 (-0.476 - 0.207)
	12	-0.132 (-0.455 - 0.221)	-0.112 (-0.454 - 0.259)	0.301 (-0.089 - 0.611)	0.182 (-0.205 - 0.520)
4-8	0	0.035 (-0.284 - 0.346)			
	1	-0.311 (-0.583 - 0.025)	-0.003 (-0.335 - 0.331)		
	2	-0.107 (-0.439 - 0.251)	0.054 (-0.300 - 0.395)	-0.160 (-0.482 - 0.200)	
	6	-0.219 (-0.528 - 0.140)	-0.183 (-0.515 - 0.196)	0.220 (-0.175 - 0.554)	-0.140 (-0.466 - 0.220)
	12	-0.207 (-0.514 - 0.147)	-0.116 (-0.457 - 0.255)	0.212 (-0.182 - 0.548)	0.245 (-0.141 - 0.566)
8-20	0	-0.117 (-0.417 - 0.206)			
	1	-0.360 (-0.619 - -0.030)*	-0.046 (-0.373 - 0.292)		
	2	-0.201 (-0.514 - 0.159)	0.263 (-0.095 - 0.560)	0.049 (-0.305 - 0.391)	
	6	0.018 (-0.332 - 0.365)	0.018 (-0.351 - 0.382)	0.292 (-0.099 - 0.605)	0.086 (-0.271 - 0.422)
	12	-0.187 (-0.498 - 0.167)	-0.151 (-0.485 - 0.222)	-0.020 (-0.397 - 0.363)	-0.199 (-0.533 - 0.188)
0-20	0	0.003 (-0.313 - 0.318)			
	1	-0.314 (-0.586 - 0.021)	-0.079 (-0.402 - 0.261)		
	2	-0.138 (-0.464 - 0.222)	0.214 (-0.146 - 0.523)	-0.089 (-0.424 - 0.268)	
	6	-0.131 (-0.459 - 0.228)	-0.166 (-0.502 - 0.214)	0.266 (-0.127 - 0.586)	-0.087 (-0.423 - 0.270)
	12	-0.160 (-0.477 - 0.194)	-0.138 (-0.474 - 0.234)	0.143 (-0.250 - 0.496)	0.012 (-0.363 - 0.383)

Ceramide depth	month	IgA (mg/ml) 0	1	2	6
0-4	0	0.068 (-0.253 - 0.375)			
	1	-0.175 (-0.480 - 0.168)	-0.363 (-0.621 - -0.034)*		
	2	-0.167 (-0.487 - 0.193)	-0.174 (-0.493 - 0.186)	-0.302 (-0.588 - 0.053)	
	6	-0.279 (-0.572 - 0.077)	-0.164 (-0.500 - 0.215)	-0.060 (-0.430 - 0.328)	-0.037 (-0.381 - 0.315)
	12	-0.167 (-0.483 - 0.187)	-0.123 (-0.463 - 0.248)	0.085 (-0.305 - 0.450)	-0.114 (-0.468 - 0.270)
4-8	0	0.060 (-0.260 - 0.369)			
	1	-0.095 (-0.415 - 0.246)	-0.218 (-0.514 - 0.125)		
	2	-0.076 (-0.414 - 0.280)	0.038 (-0.314 - 0.382)	-0.212 (-0.522 - 0.148)	
	6	-0.124 (-0.453 - 0.235)	0.005 (-0.362 - 0.371)	0.020 (-0.363 - 0.397)	-0.092 (-0.427 - 0.265)
	12	-0.157 (-0.475 - 0.197)	0.055 (-0.311 - 0.408)	0.220 (-0.175 - 0.554)	0.086 (-0.297 - 0.445)
8-20	0	0.216 (-0.107 - 0.497)			
	1	0.064 (-0.275 - 0.389)	0.117 (-0.225 - 0.433)		
	2	-0.105 (-0.438 - 0.253)	0.127 (-0.232 - 0.455)	0.023 (-0.329 - 0.369)	
	6	-0.219 (-0.527 - 0.141)	-0.150 (-0.490 - 0.229)	-0.096 (-0.460 - 0.294)	-0.076 (-0.413 - 0.281)
	12	0.041 (-0.306 - 0.379)	0.160 (-0.212 - 0.492)	0.274 (-0.118 - 0.592)	-0.114 (-0.467 - 0.271)
0-20	0	0.146 (-0.178 - 0.441)			
	1	0.063 (-0.276 - 0.388)	0.020 (-0.315 - 0.351)		
	2	-0.169 (-0.489 - 0.191)	0.019 (-0.332 - 0.365)	-0.138 (-0.464 - 0.222)	
	6	-0.178 (-0.496 - 0.182)	-0.120 (-0.466 - 0.258)	0.003 (-0.377 - 0.383)	-0.065 (-0.405 - 0.290)
	12	-0.100 (-0.429 - 0.252)	0.062 (-0.305 - 0.413)	0.198 (-0.197 - 0.537)	-0.043 (-0.409 - 0.336)

Ceramide depth	month	Lactoferrin (mg/ml) 0	1	2	6
0-4	0	0.006 (-0.310 - 0.321)			
	1	0.041 (-0.296 - 0.369)	-0.140 (-0.452 - 0.202)		
	2	-0.099 (-0.433 - 0.258)	-0.118 (-0.449 - 0.240)	-0.003 (-0.351 - 0.346)	
	6	0.015 (-0.335 - 0.362)	0.208 (-0.172 - 0.534)	-0.027 (-0.403 - 0.356)	0.085 (-0.271 - 0.422)
	12	0.018 (-0.327 - 0.359)	-0.008 (-0.367 - 0.353)	0.211 (-0.184 - 0.547)	-0.068 (-0.431 - 0.313)
4-8	0	0.030 (-0.288 - 0.342)			
	1	0.125 (-0.218 - 0.440)	-0.197 (-0.498 - 0.146)		
	2	-0.018 (-0.364 - 0.333)	-0.024 (-0.370 - 0.327)	0.059 (-0.296 - 0.399)	
	6	0.085 (-0.272 - 0.421)	0.229 (-0.150 - 0.549)	0.087 (-0.303 - 0.452)	0.154 (-0.205 - 0.477)
	12	0.069 (-0.281 - 0.403)	0.028 (-0.336 - 0.384)	0.291 (-0.101 - 0.604)	0.041 (-0.338 - 0.407)
8-20	0	-0.028 (-0.341 - 0.290)			
	1	0.038 (-0.299 - 0.367)	-0.196 (-0.496 - 0.147)		
	2	0.138 (-0.221 - 0.465)	0.246 (-0.113 - 0.547)	0.290 (-0.065 - 0.580)	
	6	0.003 (-0.346 - 0.351)	0.088 (-0.288 - 0.440)	0.107 (-0.285 - 0.468)	0.304 (-0.051 - 0.590)
	12	0.081 (-0.270 - 0.413)	-0.027 (-0.384 - 0.336)	0.166 (-0.228 - 0.514)	-0.072 (-0.434 - 0.309)
0-20	0	0.045 (-0.274 - 0.355)			
	1	0.100 (-0.241 - 0.420)	-0.198 (-0.499 - 0.144)		
	2	-0.056 (-0.397 - 0.298)	0.018 (-0.332 - 0.365)	0.119 (-0.240 - 0.449)	
	6	0.112 (-0.247 - 0.443)	0.177 (-0.203 - 0.510)	0.161 (-0.233 - 0.510)	0.232 (-0.127 - 0.537)
	12	0.140 (-0.214 - 0.461)	0.042 (-0.323 - 0.397)	0.245 (-0.148 - 0.572)	0.018 (-0.358 - 0.388)

Diff: 1.0, 0.8, 0.6, 0.4, 0.2, 0, -0.2, -0.4, -0.6, -0.8, -1.0

d

Cholesterol		Breast milk factors															
depth	month	TGF-β1 (pg./ml)				TGF-β2 (pg./ml)				IgA (mg/ml)				Lactoferrin (mg/ml)			
		0	1	2	6	0	1	2	6	0	1	2	6	0	1	2	6
0-4	0	0.031 (-0.288 - 0.343)				-0.082 (-0.387 - 0.240)				0.046 (-0.273 - 0.356)				-0.027 (-0.339 - 0.291)			
	1	0.048 (-0.290 - 0.375)	-0.016 (-0.347 - 0.319)			-0.112 (-0.429 - 0.230)	0.048 (-0.289 - 0.376)			-0.157 (-0.466 - 0.186)	-0.348 (-0.610 - -0.017)*			-0.048 (-0.375 - 0.290)	-0.124 (-0.439 - 0.219)		
	2	0.261 (-0.096 - 0.559)	0.013 (-0.337 - 0.360)	-0.065 (-0.404 - 0.291)		0.091 (-0.266 - 0.426)	-0.214 (-0.524 - 0.145)	-0.451 (-0.691 - -0.121)*		0.103 (-0.254 - 0.436)	-0.021 (-0.367 - 0.330)	-0.161 (-0.483 - 0.199)		-0.111 (-0.443 - 0.247)	-0.040 (-0.383 - 0.313)	-0.246 (-0.548 - 0.112)	
	6	-0.221 (-0.529 - 0.138)	-0.410 (-0.675 - -0.052)*	-0.475 (-0.724 - -0.116)*	-0.422 (-0.672 - -0.086)*	-0.401 (-0.658 - -0.061)*	-0.281 (-0.587 - 0.096)	-0.051 (-0.423 - 0.335)	-0.414 (-0.666 - -0.076)*	0.275 (-0.082 - 0.569)	0.165 (-0.214 - 0.501)	0.201 (-0.194 - 0.540)	0.049 (-0.305 - 0.391)	0.030 (-0.322 - 0.375)	-0.098 (-0.448 - 0.278)	-0.059 (-0.429 - 0.329)	-0.361 (-0.630 - -0.014)*
	12	-0.054 (-0.390 - 0.294)	-0.291 (-0.590 - 0.077)	-0.203 (-0.541 - 0.192)	-0.355 (-0.643 - 0.021)	-0.051 (-0.387 - 0.298)	-0.224 (-0.541 - 0.148)	-0.070 (-0.439 - 0.318)	-0.165 (-0.507 - 0.222)	0.030 (-0.316 - 0.370)	-0.194 (-0.518 - 0.179)	-0.073 (-0.441 - 0.316)	-0.396 (-0.670 - -0.027)*	-0.033 (-0.372 - 0.314)	-0.037 (-0.392 - 0.328)	0.095 (-0.296 - 0.458)	-0.362 (-0.647 - 0.013)
4-8	0	-0.087 (-0.392 - 0.235)				-0.135 (-0.432 - 0.189)				0.065 (-0.256 - 0.373)				0.060 (-0.261 - 0.368)			
	1	-0.025 (-0.356 - 0.310)	-0.135 (-0.448 - 0.208)			-0.137 (-0.450 - 0.206)	0.045 (-0.293 - 0.372)			-0.161 (-0.469 - 0.183)	-0.313 (-0.585 - 0.022)			0.039 (-0.298 - 0.367)	-0.183 (-0.487 - 0.160)		
	2	0.352 (0.003 - 0.624)*	0.014 (-0.336 - 0.361)	-0.152 (-0.476 - 0.208)		0.154 (-0.205 - 0.477)	-0.212 (-0.522 - 0.148)	-0.449 (-0.690 - -0.119)*		0.083 (-0.274 - 0.419)	-0.092 (-0.427 - 0.265)	-0.156 (-0.478 - 0.204)		-0.206 (-0.517 - 0.154)	-0.197 (-0.511 - 0.163)	-0.258 (-0.557 - 0.100)	
	6	-0.025 (-0.370 - 0.327)	-0.199 (-0.527 - 0.181)	-0.317 (-0.622 - 0.071)	-0.340 (-0.616 - 0.010)	-0.362 (-0.631 - -0.015)*	-0.144 (-0.485 - 0.235)	-0.112 (-0.472 - 0.280)	-0.352 (-0.624 - -0.003)*	0.051 (-0.303 - 0.393)	0.033 (-0.338 - 0.394)	0.033 (-0.351 - 0.408)	-0.085 (-0.422 - 0.271)	-0.199 (-0.512 - 0.161)	-0.180 (-0.513 - 0.200)	-0.349 (-0.644 - 0.036)	-0.254 (-0.554 - 0.104)
	12	-0.064 (-0.398 - 0.286)	-0.098 (-0.443 - 0.272)	0.065 (-0.323 - 0.435)	-0.161 (-0.504 - 0.225)	0.048 (-0.300 - 0.385)	-0.051 (-0.403 - 0.316)	0.095 (-0.295 - 0.459)	-0.118 (-0.470 - 0.267)	0.045 (-0.303 - 0.382)	0.146 (-0.226 - 0.481)	0.098 (-0.293 - 0.461)	-0.224 (-0.551 - 0.163)	0.286 (-0.063 - 0.573)	0.112 (-0.258 - 0.454)	0.198 (-0.196 - 0.538)	-0.206 (-0.538 - 0.181)
8-20	0	0.089 (-0.233 - 0.394)				0.049 (-0.270 - 0.359)				0.229 (-0.094 - 0.508)				0.356 (0.045 - 0.604)*			
	1	-0.027 (-0.357 - 0.309)	-0.106 (-0.424 - 0.236)			-0.040 (-0.369 - 0.297)	0.090 (-0.250 - 0.411)			0.013 (-0.321 - 0.345)	0.107 (-0.235 - 0.425)			0.317 (-0.018 - 0.588)	-0.163 (-0.471 - 0.180)		
	2	0.047 (-0.307 - 0.389)	0.038 (-0.314 - 0.382)	-0.017 (-0.364 - 0.333)		-0.166 (-0.487 - 0.194)	-0.011 (-0.359 - 0.339)	-0.192 (-0.507 - 0.167)		0.131 (-0.228 - 0.459)	-0.049 (-0.391 - 0.305)	0.049 (-0.305 - 0.391)		0.012 (-0.338 - 0.360)	-0.079 (-0.416 - 0.277)	0.039 (-0.314 - 0.382)	
	6	-0.024 (-0.370 - 0.327)	-0.178 (-0.511 - 0.202)	-0.025 (-0.401 - 0.358)	0.064 (-0.291 - 0.404)	-0.192 (-0.507 - 0.168)	-0.276 (-0.584 - 0.100)	-0.021 (-0.398 - 0.362)	-0.041 (-0.384 - 0.312)	0.031 (-0.321 - 0.375)	-0.069 (-0.425 - 0.305)	-0.032 (-0.407 - 0.352)	0.085 (-0.272 - 0.421)	0.069 (-0.287 - 0.408)	-0.090 (-0.442 - 0.286)	-0.077 (-0.444 - 0.312)	-0.061 (-0.401 - 0.294)
	12	0.235 (-0.118 - 0.535)	-0.102 (-0.446 - 0.268)	-0.036 (-0.410 - 0.349)	-0.245 (-0.566 - 0.141)	0.125 (-0.228 - 0.449)	-0.185 (-0.511 - 0.188)	-0.059 (-0.429 - 0.329)	-0.305 (-0.609 - 0.076)	0.282 (-0.067 - 0.570)	0.146 (-0.226 - 0.481)	0.379 (-0.002 - 0.663)	-0.224 (-0.551 - 0.163)	0.148 (-0.205 - 0.468)	-0.002 (-0.362 - 0.358)	0.275 (-0.117 - 0.593)	-0.376 (-0.657 - -0.003)*
0-20	0	0.076 (-0.245 - 0.382)				-0.076 (-0.382 - 0.246)				0.184 (-0.140 - 0.472)				0.242 (-0.079 - 0.518)			
	1	0.017 (-0.318 - 0.348)	0.005 (-0.329 - 0.337)			-0.147 (-0.458 - 0.196)	0.067 (-0.272 - 0.392)			-0.027 (-0.357 - 0.309)	-0.109 (-0.426 - 0.233)			0.191 (-0.152 - 0.493)	-0.149 (-0.459 - 0.194)		
	2	0.213 (-0.147 - 0.523)	0.015 (-0.335 - 0.362)	-0.103 (-0.436 - 0.255)		-0.052 (-0.393 - 0.302)	-0.175 (-0.494 - 0.185)	-0.402 (-0.658 - -0.062)*		0.040 (-0.313 - 0.383)	-0.084 (-0.421 - 0.272)	-0.091 (-0.426 - 0.266)		-0.168 (-0.488 - 0.192)	-0.183 (-0.500 - 0.177)	-0.251 (-0.552 - 0.107)	
	6	-0.037 (-0.381 - 0.316)	-0.119 (-0.465 - 0.259)	-0.201 (-0.540 - 0.194)	-0.109 (-0.441 - 0.250)	-0.332 (-0.610 - 0.019)	-0.191 (-0.521 - 0.189)	0.036 (-0.349 - 0.410)	-0.124 (-0.453 - 0.235)	0.100 (-0.258 - 0.433)	-0.092 (-0.444 - 0.284)	-0.106 (-0.467 - 0.286)	0.011 (-0.339 - 0.358)	0.042 (-0.311 - 0.385)	-0.147 (-0.487 - 0.232)	-0.132 (-0.487 - 0.261)	-0.190 (-0.505 - 0.170)
	12	0.189 (-0.165 - 0.500)	-0.065 (-0.416 - 0.302)	0.034 (-0.350 - 0.409)	-0.286 (-0.596 - 0.097)	0.095 (-0.256 - 0.425)	-0.192 (-0.517 - 0.181)	0.037 (-0.348 - 0.411)	-0.311 (-0.613 - 0.070)	0.196 (-0.158 - 0.505)	0.043 (-0.322 - 0.397)	0.262 (-0.131 - 0.584)	-0.359 (-0.646 - 0.016)	0.135 (-0.218 - 0.457)	0.028 (-0.336 - 0.384)	0.273 (-0.120 - 0.592)	-0.448 (-0.703 - -0.090)*

Diff
1.0
0.8
0.6
0.4
0.2
0
-0.2
-0.4
-0.6
-0.8
-1.0

e

Lactic acid		Breast milk factors															
depth	month	TGF-β1 (pg./ml)				TGF-β2 (pg./ml)				IgA (mg/ml)				Lactoferrin (mg/ml)			
		0	1	2	6	0	1	2	6	0	1	2	6	0	1	2	6
0-4	0	0.283 (-0.035 - 0.550)				0.081 (-0.241 - 0.386)				0.259 (-0.062 - 0.531)				-0.065 (-0.373 - 0.256)			
	1	-0.111 (-0.428 - 0.231)	-0.091 (-0.412 - 0.250)			-0.263 (-0.548 - 0.077)	0.064 (-0.275 - 0.389)			0.209 (-0.133 - 0.507)	0.345 (0.013 - 0.608)*			0.129 (-0.214 - 0.443)	0.192 (-0.151 - 0.493)		
	2	-0.022 (-0.368 - 0.329)	0.218 (-0.142 - 0.526)	0.269 (-0.087 - 0.565)		-0.238 (-0.542 - 0.121)	0.201 (-0.159 - 0.514)	0.158 (-0.202 - 0.480)		-0.131 (-0.459 - 0.228)	0.161 (-0.199 - 0.483)	0.085 (-0.272 - 0.421)		0.071 (-0.285 - 0.409)	-0.141 (-0.467 - 0.218)	0.047 (-0.307 - 0.389)	
	6	0.303 (-0.051 - 0.589)	-0.027 (-0.390 - 0.343)	0.098 (-0.293 - 0.461)	-0.094 (-0.429 - 0.263)	0.238 (-0.121 - 0.541)	0.092 (-0.284 - 0.444)	0.270 (-0.123 - 0.589)	0.162 (-0.198 - 0.483)	-0.030 (-0.375 - 0.322)	-0.001 (-0.368 - 0.365)	0.172 (-0.222 - 0.518)	-0.061 (-0.401 - 0.294)	0.088 (-0.269 - 0.424)	-0.112 (-0.459 - 0.266)	0.156 (-0.238 - 0.506)	0.040 (-0.313 - 0.383)
	12	-0.173 (-0.488 - 0.181)	0.100 (-0.270 - 0.444)	0.305 (-0.085 - 0.614)	0.285 (-0.098 - 0.595)	-0.167 (-0.482 - 0.187)	0.036 (-0.329 - 0.391)	0.393 (0.015 - 0.672)*	0.180 (-0.207 - 0.518)	0.007 (-0.337 - 0.350)	0.262 (-0.108 - 0.569)	0.319 (-0.070 - 0.623)	0.322 (-0.058 - 0.620)	0.353 (0.011 - 0.621)*	0.349 (-0.013 - 0.630)	0.343 (-0.042 - 0.640)	0.333 (-0.045 - 0.628)
4-8	0	0.213 (-0.110 - 0.495)				0.051 (-0.269 - 0.360)				0.257 (-0.064 - 0.530)				-0.127 (-0.425 - 0.197)			
	1	-0.144 (-0.455 - 0.199)	-0.159 (-0.468 - 0.184)			-0.208 (-0.506 - 0.134)	0.024 (-0.312 - 0.354)			0.321 (-0.014 - 0.591)	0.450 (0.138 - 0.681)*			0.314 (-0.021 - 0.586)	0.188 (-0.155 - 0.491)		
	2	-0.064 (-0.404 - 0.291)	0.204 (-0.156 - 0.516)	0.249 (-0.109 - 0.550)		-0.179 (-0.496 - 0.181)	0.087 (-0.270 - 0.422)	0.232 (-0.127 - 0.538)		0.111 (-0.247 - 0.443)	0.297 (-0.057 - 0.585)	0.173 (-0.187 - 0.492)		0.178 (-0.182 - 0.496)	-0.003 (-0.351 - 0.346)	0.000 (-0.348 - 0.349)	
	6	0.332 (-0.019 - 0.610)	0.051 (-0.322 - 0.410)	0.139 (-0.255 - 0.493)	-0.089 (-0.424 - 0.268)	0.227 (-0.133 - 0.533)	0.115 (-0.263 - 0.462)	0.328 (-0.059 - 0.630)	0.163 (-0.196 - 0.485)	-0.018 (-0.364 - 0.333)	-0.007 (-0.372 - 0.361)	0.118 (-0.274 - 0.477)	-0.046 (-0.388 - 0.308)	0.012 (-0.338 - 0.359)	-0.146 (-0.487 - 0.233)	0.112 (-0.280 - 0.472)	0.030 (-0.322 - 0.375)
	12	-0.129 (-0.453 - 0.224)	0.045 (-0.320 - 0.399)	0.255 (-0.138 - 0.579)	0.194 (-0.193 - 0.529)	-0.066 (-0.400 - 0.284)	0.030 (-0.334 - 0.386)	0.312 (-0.077 - 0.619)	0.036 (-0.342 - 0.403)	0.194 (-0.160 - 0.503)	0.370 (0.011 - 0.644)*	0.494 (0.140 - 0.736)*	0.267 (-0.118 - 0.582)	0.404 (0.070 - 0.656)*	0.314 (-0.052 - 0.606)	0.376 (-0.005 - 0.662)	0.151 (-0.236 - 0.496)
8-20	0	-0.050 (-0.360 - 0.269)				-0.089 (-0.394 - 0.233)				0.170 (-0.154 - 0.461)				0.033 (-0.286 - 0.345)			
	1	0.161 (-0.183 - 0.469)	0.061 (-0.278 - 0.386)			0.087 (-0.254 - 0.408)	0.076 (-0.264 - 0.399)			-0.076 (-0.399 - 0.264)	-0.097 (-0.417 - 0.244)			-0.023 (-0.354 - 0.312)	0.095 (-0.246 - 0.415)		
	2	0.177 (-0.183 - 0.495)	0.276 (-0.080 - 0.570)	0.485 (0.164 - 0.713)*		0.013 (-0.337 - 0.360)	0.293 (-0.062 - 0.582)	0.291 (-0.065 - 0.581)		-0.026 (-0.371 - 0.326)	-0.294 (-0.583 - 0.061)	-0.078 (-0.415 - 0.278)		0.147 (-0.212 - 0.472)	0.005 (-0.344 - 0.354)	0.111 (-0.248 - 0.442)	
	6	0.166 (-0.194 - 0.487)	-0.172 (-0.507 - 0.208)	0.020 (-0.363 - 0.397)	0.024 (-0.328 - 0.369)	0.293 (-0.063 - 0.582)	0.039 (-0.332 - 0.400)	0.154 (-0.240 - 0.504)	0.161 (-0.199 - 0.483)	0.191 (-0.169 - 0.506)	0.219 (-0.161 - 0.542)	0.418 (0.045 - 0.688)*	0.176 (-0.184 - 0.495)	0.183 (-0.177 - 0.500)	-0.097 (-0.448 - 0.279)	0.175 (-0.219 - 0.521)	-0.104 (-0.437 - 0.254)
	12	-0.192 (-0.502 - 0.162)	-0.048 (-0.402 - 0.318)	0.163 (-0.231 - 0.511)	0.187 (-0.200 - 0.524)	-0.189 (-0.500 - 0.165)	-0.100 (-0.445 - 0.270)	0.058 (-0.329 - 0.429)	-0.129 (-0.479 - 0.257)	0.168 (-0.186 - 0.483)	0.380 (0.023 - 0.651)*	0.618 (0.311 - 0.808)*	0.200 (-0.187 - 0.534)	0.165 (-0.189 - 0.481)	0.123 (-0.249 - 0.462)	0.240 (-0.154 - 0.568)	-0.157 (-0.500 - 0.230)
0-20	0	0.142 (-0.181 - 0.438)				0.033 (-0.285 - 0.345)				0.172 (-0.152 - 0.462)				0.006 (-0.310 - 0.321)			
	1	-0.010 (-0.342 - 0.325)	-0.048 (-0.376 - 0.289)			-0.133 (-0.447 - 0.209)	0.059 (-0.279 - 0.385)			0.174 (-0.169 - 0.479)	0.161 (-0.182 - 0.469)			0.173 (-0.170 - 0.479)	0.112 (-0.230 - 0.429)		
	2	0.085 (-0.272 - 0.421)	0.347 (-0.002 - 0.621)	0.545 (0.242 - 0.751)*		-0.074 (-0.412 - 0.282)	0.288 (-0.067 - 0.579)	0.404 (0.064 - 0.660)*		0.007 (-0.343 - 0.355)	-0.038 (-0.382 - 0.315)	0.065 (-0.291 - 0.404)		0.183 (-0.177 - 0.500)	0.037 (-0.316 - 0.380)	0.165 (-0.195 - 0.485)	
	6	0.317 (-0.036 - 0.599)	0.092 (-0.284 - 0.443)	0.163 (-0.231 - 0.511)	0.013 (-0.337 - 0.360)	0.266 (-0.091 - 0.562)	0.210 (-0.169 - 0.536)	0.322 (-0.066 - 0.625)	0.269 (-0.087 - 0.565)	-0.082 (-0.419 - 0.275)	0.045 (-0.327 - 0.405)	0.153 (-0.241 - 0.504)	0.036 (-0.316 - 0.380)	0.055 (-0.300 - 0.396)	-0.086 (-0.438 - 0.290)	0.108 (-0.284 - 0.469)	0.106 (-0.252 - 0.438)
	12	-0.159 (-0.477 - 0.195)	-0.016 (-0.374 - 0.346)	0.216 (-0.179 - 0.551)	0.152 (-0.235 - 0.497)	-0.229 (-0.531 - 0.124)	-0.089 (-0.435 - 0.280)	0.184 (-0.211 - 0.527)	-0.102 (-0.458 - 0.282)	0.156 (-0.198 - 0.474)	0.342 (-0.020 - 0.625)	0.573 (0.247 - 0.783)*	0.195 (-0.192 - 0.530)	0.269 (-0.082 - 0.561)	0.227 (-0.145 - 0.543)	0.300 (-0.090 - 0.611)	-0.031 (-0.399 - 0.346)

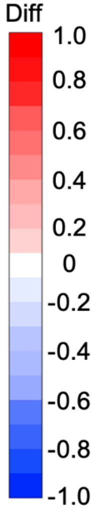

Figure S5

MPA	month	Breast milk factors															
		TGF-β1 (pg./ml)				TGF-β2 (pg./ml)				IgA (mg/ml)				Lactoferrin (mg/ml)			
		0	1	2	6	0	1	2	6	0	1	2	6	0	1	2	6
SC hydration (a.u.)	0	0.058 (-0.262 - 0.367)				-0.146 (-0.442 - 0.177)				-0.122 (-0.421 - 0.202)				-0.380 (-0.621 - -0.074)*			
	1	0.166 (-0.177 - 0.473)	0.065 (-0.275 - 0.389)			-0.166 (-0.473 - 0.177)	0.055 (-0.284 - 0.381)			-0.485 (-0.704 - -0.181)*	-0.465 (-0.691 - -0.156)*			-0.547 (-0.745 - -0.262)*	-0.407 (-0.652 - -0.085)*		
	2	0.518 (0.207 - 0.734)*	0.422 (0.086 - 0.672)*	0.453 (0.124 - 0.693)*		0.173 (-0.187 - 0.492)	0.203 (-0.157 - 0.515)	0.344 (-0.005 - 0.619)		-0.271 (-0.567 - 0.085)	-0.308 (-0.593 - 0.045)	-0.266 (-0.563 - 0.091)		0.113 (-0.246 - 0.444)	-0.184 (-0.500 - 0.176)	0.192 (-0.168 - 0.507)	
	6	0.091 (-0.266 - 0.426)	-0.019 (-0.383 - 0.350)	0.038 (-0.347 - 0.412)	-0.025 (-0.371 - 0.326)	0.098 (-0.259 - 0.432)	0.053 (-0.320 - 0.412)	0.225 (-0.169 - 0.558)	0.009 (-0.341 - 0.356)	0.104 (-0.254 - 0.437)	0.143 (-0.236 - 0.484)	0.222 (-0.172 - 0.555)	0.118 (-0.241 - 0.448)	-0.218 (-0.527 - 0.141)	-0.153 (-0.492 - 0.227)	-0.106 (-0.467 - 0.286)	-0.121 (-0.451 - 0.238)
	12	-0.021 (-0.362 - 0.325)	-0.295 (-0.592 - 0.073)	-0.130 (-0.486 - 0.263)	-0.187 (-0.524 - 0.200)	0.012 (-0.333 - 0.354)	-0.301 (-0.597 - 0.066)	-0.136 (-0.491 - 0.257)	-0.270 (-0.585 - 0.114)	-0.099 (-0.428 - 0.253)	-0.188 (-0.513 - 0.185)	-0.283 (-0.598 - 0.109)	-0.027 (-0.396 - 0.349)	0.026 (-0.320 - 0.366)	-0.205 (-0.526 - 0.168)	-0.133 (-0.488 - 0.260)	0.152 (-0.234 - 0.497)
TEWL (g/h/m2)	0	-0.170 (-0.461 - 0.154)				-0.294 (-0.558 - 0.024)				0.102 (-0.221 - 0.404)				0.074 (-0.247 - 0.381)			
	1	0.052 (-0.286 - 0.379)	-0.029 (-0.359 - 0.307)			-0.272 (-0.555 - 0.067)	-0.015 (-0.346 - 0.320)			-0.357 (-0.617 - -0.027)*	-0.436 (-0.671 - -0.120)*			-0.068 (-0.392 - 0.272)	-0.190 (-0.492 - 0.153)		
	2	0.014 (-0.337 - 0.361)	0.113 (-0.245 - 0.444)	0.315 (-0.038 - 0.598)		0.088 (-0.269 - 0.423)	0.271 (-0.086 - 0.566)	0.216 (-0.143 - 0.525)		-0.024 (-0.369 - 0.328)	-0.274 (-0.568 - 0.083)	0.070 (-0.286 - 0.409)		0.195 (-0.165 - 0.509)	0.009 (-0.341 - 0.356)	0.260 (-0.097 - 0.558)	
	6	-0.137 (-0.463 - 0.223)	-0.255 (-0.569 - 0.123)	0.246 (-0.148 - 0.573)	-0.310 (-0.595 - 0.043)	0.048 (-0.305 - 0.390)	-0.202 (-0.529 - 0.178)	0.098 (-0.293 - 0.461)	-0.301 (-0.588 - 0.053)	-0.294 (-0.583 - 0.061)	-0.257 (-0.570 - 0.121)	-0.022 (-0.399 - 0.361)	-0.335 (-0.612 - 0.016)	0.118 (-0.240 - 0.449)	0.242 (-0.136 - 0.559)	-0.043 (-0.416 - 0.343)	-0.063 (-0.403 - 0.292)
	12	-0.158 (-0.476 - 0.196)	0.062 (-0.305 - 0.413)	0.194 (-0.201 - 0.535)	0.014 (-0.361 - 0.385)	-0.059 (-0.394 - 0.290)	0.107 (-0.263 - 0.450)	0.216 (-0.179 - 0.550)	0.044 (-0.334 - 0.411)	0.054 (-0.295 - 0.390)	0.299 (-0.068 - 0.595)	0.264 (-0.129 - 0.585)	0.062 (-0.318 - 0.426)	0.378 (0.039 - 0.638)*	0.248 (-0.123 - 0.559)	0.527 (0.184 - 0.756)*	0.271 (-0.113 - 0.585)

Diff
1.0
0.8
0.6
0.4
0.2
0
-0.2
-0.4
-0.6
-0.8
-1.0

Figure S5 Spearman's Correlation coefficient between stratum corneum (SC) hydration and transepidermal water loss (TEWL) in the SC and breast milk factors at each month of age

Correlation coefficients and 95% confidence intervals between the median of SC hydration and TEWL and breast milk factors (TGF-β1.2, IgA, lactoferrin) at each month of age are shown; p< 0.05 * . For SC hydration, TGF-β1 and 2 levels correlated positively for almost the entire period, while IgA and lactoferrin correlated negatively. TEWL was partially positively correlated in all breast milk factors.

Figure S6

SC thickness	Breast milk factors							
month	TGF-β1 (pg./ml)				TGF-β2 (pg./ml)			
	0	1	2	6	0	1	2	6
0	0.069 (-0.252 - 0.376)				0.051 (-0.269 - 0.361)			
1	-0.150 (-0.460 - 0.193)	-0.140 (-0.452 - 0.203)			-0.019 (-0.350 - 0.316)	-0.101 (-0.420 - 0.241)		
2	-0.130 (-0.458 - 0.229)	-0.067 (-0.406 - 0.288)	-0.207 (-0.519 - 0.152)		-0.290 (-0.580 - 0.066)	0.069 (-0.286 - 0.408)	-0.023 (-0.369 - 0.328)	
6	0.375 (0.030 - 0.640)*	0.322 (-0.051 - 0.616)	0.513 (0.165 - 0.747)*	0.057 (-0.298 - 0.398)	0.425 (0.090 - 0.674)*	0.070 (-0.304 - 0.426)	0.373 (-0.008 - 0.660)	0.169 (-0.191 - 0.489)
12	0.053 (-0.296 - 0.389)	0.147 (-0.225 - 0.482)	0.167 (-0.227 - 0.515)	0.169 (-0.218 - 0.510)	-0.065 (-0.399 - 0.285)	0.032 (-0.332 - 0.388)	0.015 (-0.367 - 0.392)	0.274 (-0.111 - 0.587)

SC thickness	Breast milk factors							
month	IgA (mg/ml)				Lactoferrin (mg/ml)			
	0	1	2	6	0	1	2	6
0	0.210 (-0.113 - 0.493)				0.288 (-0.030 - 0.553)			
1	0.009 (-0.325 - 0.341)	0.158 (-0.185 - 0.467)			0.279 (-0.060 - 0.560)	0.089 (-0.252 - 0.410)		
2	0.010 (-0.340 - 0.357)	0.180 (-0.180 - 0.497)	0.276 (-0.080 - 0.570)		0.026 (-0.326 - 0.371)	-0.205 (-0.517 - 0.154)	0.095 (-0.262 - 0.429)	
6	0.052 (-0.302 - 0.393)	-0.118 (-0.464 - 0.260)	-0.083 (-0.449 - 0.307)	-0.117 (-0.447 - 0.242)	0.271 (-0.086 - 0.566)	0.195 (-0.185 - 0.524)	0.217 (-0.177 - 0.552)	0.165 (-0.195 - 0.486)
12	0.251 (-0.101 - 0.547)	0.099 (-0.271 - 0.443)	0.266 (-0.127 - 0.586)	0.234 (-0.153 - 0.558)	0.204 (-0.150 - 0.511)	0.243 (-0.128 - 0.555)	0.297 (-0.094 - 0.608)	0.431 (0.069 - 0.693)*

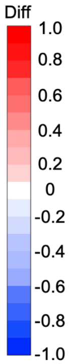

Figure S6 Spearman’s Correlation coefficient between the stratum corneum (SC) thickness and breast milk factors at each month of age

Correlation coefficients and 95% confidence intervals between SC thickness and breast milk factors (TGF-β1.2, IgA, lactoferrin) at each month of age are shown; $p< 0.05$＊. Over the study period, SC thickness showed a weak to moderate positive correlation with all breast milk factors. In particular, TGF-β1 at 2 months showed a strong positive correlation with SC thickness at 6 months.

Table S1

(a)

range of AUC	median [IQR] (mass-%.µm)				
	0M (N = 39)	1M (N = 35)	2M (N = 32)	6M (N = 32)	12M (N = 33)
0-4	98.774 [87.839-108.885]	130.938 [119.582-137.989]	136.712 [124.328-143.826]	134.38 [128.646-140.47]	121.248 [112.493-131.966]
4-8	121.682 [109.493-129.3]	158.216 [150.505-163.938]	166.538 [157.903-174.148]	158.517 [149.299-168.299]	147.766 [135.482-155.98]
8-20	537.336 [498.841-583.705]	680.848 [653.007-704.119]	705.204 [680.678-728.045]	668.872 [629.568-692.221]	641.244 [611.38-672.382]
0-20	754.496 [690.043-822.31]	967.122 [934.946-995.892]	1012.503 [980.74-1040.574]	967.709 [912.031-989.166]	901.256 [855.624-955.656]

(b)

range of AUC	median [IQR] (a.u.・µm)				
	0M (N = 39)	1M (N = 35)	2M (N = 32)	6M (N = 32)	12M (N = 33)
0-4	2.067 [1.364-2.883]	3.186 [2.473-3.762]	1.997 [1.616-2.99]	2.365 [1.278-3.414]	2.811 [2.298-3.236]
4-8	3.032 [2.478-3.642]	3.17 [2.598-3.741]	1.857 [1.483-3.021]	2.272 [1.698-3.278]	3.016 [2.177-3.806]
8-20	7.429 [5.13-9.742]	2.871 [1.931-4.063]	1.787 [1.137-3.199]	2.718 [1.926-3.687]	3.189 [2.284-4.75]
0-20	11.867 [9.572-15.766]	9.486 [7.439-11.031]	5.844 [4.615-9.088]	8.04 [4.985-10.557]	8.755 [6.595-12.247]

(c)

range of AUC	median [IQR] (a.u.・µm)				
	0M (N = 39)	1M (N = 35)	2M (N = 32)	6M (N = 32)	12M (N = 33)
0-4	642.2 [560.29-764.1]	385.908 [338.883-416.536]	326.85 [276.842-380.234]	232.383 [206.315-274.788]	230.97 [196.782-295.768]
4-8	599.7 [538.42-682.36]	352.956 [316.928-385.804]	274.888 [236.385-312.857]	212.933 [186.274-247.859]	201.62 [170.09-241.318]
8-20	1444.028 [1292.817-1544.632]	707.18 [697.152-822.14]	545.656 [501.105-605.218]	466.677 [406.466-567.08]	425.24 [391.466-555.095]
0-20	2687.16 [2448.113-3017.41]	1468.368 [1347.428-1631.503]	1153.214 [1043.878-1271.735]	863.678 [788.576-1146.083]	869.486 [774.484-1078.822]

(d)

range of AUC	median [IQR] (a.u.・µm)				
	0M (N = 39)	1M (N = 35)	2M (N = 32)	6M (N = 32)	12M (N = 33)
0-4	0.3 [0.273-0.357]	0.219 [0.198-0.259]	0.179 [0.166-0.21]	0.139 [0.12-0.154]	0.124 [0.105-0.152]
4-8	0.291 [0.261-0.33]	0.205 [0.174-0.222]	0.157 [0.132-0.182]	0.123 [0.099-0.137]	0.113 [0.1-0.133]
8-20	0.68 [0.604-0.735]	0.362 [0.316-0.383]	0.3 [0.253-0.341]	0.235 [0.22-0.292]	0.258 [0.2-0.313]
0-20	1.291 [1.158-1.489]	0.807 [0.706-0.861]	0.644 [0.584-0.709]	0.537 [0.457-0.579]	0.522 [0.417-0.578]

(e)

range of AUC	median [IQR] (a.u.・µm)				
	0M (N = 39)	1M (N = 35)	2M (N = 32)	6M (N = 32)	12M (N = 33)
0-4	120.308 [105.35-167.062]	109.362 [89.752-129.095]	125.069 [102.058-144.426]	184.728 [141.705-247.682]	167.168 [127.332-199.712]
4-8	77.996 [66.478-103.454]	67.862 [54.214-81.91]	76.54 [66.567-87.428]	117.634 [91.23-168.997]	108.436 [72.12-137.262]
8-20	169.28 [145.79-204.078]	184.371 [153.956-221.005]	200.975 [167.244-236.37]	240.077 [216.165-315.193]	232.552 [179.07-290.88]
0-20	374.638 [321.099-445.874]	370.578 [316.986-418.003]	409.407 [366.764-457.401]	537.829 [454.906-722.808]	476.018 [409.067-639.652]

Table S1 Median values and interquartile range (IQR) for median area under the curve (AUC) of molecular components in the stratum corneum (SC) as measured by confocal Raman spectrometry by depth for each age

Median values and interquartile range (IQR) for AUC of molecular components for water content (a), NMF (b), ceramide (c), cholesterol (d), and lactic acid (e) at depths of 0-4, 4-8, 8-20, and 0-20 µm of the SC, respectively.

Table S2

median [IQR]	0M (N = 39)	1M (N = 35)	2M (N = 32)	6M (N = 32)	12M (N = 33)
SC thickness (μm)	22.485 [21.093-25.440]	17.222 [14.984-17.984]	14.322 [13.571-16.964]	17.358 [14.535-18.553]	17.38 [14.577-21.262]

Table S2 Median values and interquartile range (IQR) of the SC thickness

Median values and IQR for the SC thickness are shown.

Table S3

median [IQR]	0M (N = 39)	1M (N = 35)	2M (N = 32)	6M (N = 32)	12M (N = 33)
SC hydration (a.u.)	26.2 [20.25-30.45]	51.9 [40.75-59.2]	55.55 [47.75-65.475]	56.25 [47.5-68.05]	43.4 [35.2-47.3]
TEWL(g/h/m^2)	13.94 [10.575-17.485]	14.29 [11.625-17.87]	22.5 [18.312-27.443]	23.3 [17.972-27.77]	20.26 [17.155-23.74]

Table S3 Median values and interquartile range (IQR) of stratum corneum (SC) hydration and transepidermal water loss (TEWL)

Median values and interquartile range (IQR) of AUC of SC hydration and TEWL are shown.

Table S4

median [IQR]	0M (N = 39)	1M (N = 35)	2M (N = 32)	6M (N = 32)
TGF-β1 (pg/ml)	1027.064 [776.483-1720.352]	479.708 [350.432-585.14]	485.985 [358.528-595.772]	424.615 [347.424-634.226]
TGF-β2 (pg/ml)	5321.776 [3880.672-10496.838]	1210.482 [871.732-2193.36]	1289.574 [853.082-2277.596]	2324.213 [1087.839-6893.642]
IgA (mg/ml)	3.594 [2.08-8.514]	0.882 [0.568-1.329]	0.611 [0.488-0.873]	0.543 [0.408-0.783]
Lactoferrin (mg/ml)	2.146 [1.644-3.206]	0.585 [0.448-1.085]	0.341 [0.321-0.45]	0.207 [0.165-0.326]

Table S4 Median values and interquartile range (IQR) for breast milk factors

Median values and IQR for TGF-β1, TGF-β2, IgA, and lactoferrin in breast milk by age in months.
